# Facts for and against

**Published:** 1921-04-23

**Authors:** 


### THE CUSTOM OF DRINKING TEA.

### Facts For and Against.

The custom of tea drinking is so universal and its usual ill-effects so insignificant that it may seem an unnecessary exaggeration to speak of tea intoxication. Yet, without applauding the outpourings of cranks upon the subject, the medical profession will do well to consider the point. Professor Allen Starr, of Columbia University, gives, in a recent communication to the *Medical Record*, a highly interesting analysis of the possibilities, and brings to light many facts we are apt to overlook.

It is not always appreciated that, next to water, tea is the most widely used beverage in the world. The age of the custom can be judged from its recorded prevalence in China as early as 2,700 B.C., although it was apparently not brought into Europe until A.D. 1600. At this period it was of necessity a luxury, and sold, in fact, at £10 per pound! Of its rarity one may read at 1660 in Pepys' diary: "I did send for a cup of tea, a China drink which I had never drunk before." A little later, however, the East India Company began to import it freely into England, and to-day statistics tell us that for the average person in the British Empire six pounds of tea are annually required. Within the last few years most countries show a definite increase in the tea-drinking proportion of their population, and in the United States there has, in five years, been a rise of 40 per cent. in the actual amount imported.

### Tea Intoxication.

But to return to tea intoxication, as Professor Starr explains, the acute condition is extremely rare. He can find only one case in medical literature, and that in the instance of a woman who, by misunderstanding some professional advice, took 300 grams of tea in 300 c.c. of water. This quantity represented about 9 grams, or 135 grains, of pure caffeine (the active principle of tea), and although not actually fatal, the symptoms were those of acute caffeine poisoning, with vomiting and complete prostration.

A rare instance of this kind need not, however, detain us. The effect of sub-acute intoxication is far commoner and chiefly upon the circulatory and vasomotor systems. Sir James Mackenzie, to instance the susceptibility of the body to the constituents of tea, quotes the case of a man who, after an attack of influenza, could not drink a cup of tea without his pulse intermitting every third or fourth beat for a noticeable time. This tendency persisted for several months, and such cases are by no means uncommon. At least, they remind us that even one cup of tea does contain an appreciable amount of alkaloid. Cases where tea equivalent to 5-10 grains of caffeine has been taken show that mental acuity is at first decidedly stimulated, but some two hours later a physical weakness develops, the mind grows uneasy and ultimately an obstinate sleeplessness persists. That such poisonings are not uncommon and occur indeed in most unexpected circumstances is well instanced by an example from Professor Starr's own experience.

Some time ago the captain of a college football team brought three of its members to him in a state of great nervousness. These men were all strong and characteristically fit, but now suffered from marked tremour of the hands, restlessness, insomnia, and loss of appetite and indigestion, in spite of the careful dietary at the "training-table." Depression and nervous apprehension were extraordinarily marked, and they had already been treated for two weeks by a physician, with tonics containing strychnine. The captain, having seen the prescription, thought they were being poisoned by strychnine purposely given by the physician, who was a graduate of the rival college! The strychnine dose given was, however, $\frac{1}{60}$ gr. only., quite inadequate to produce the symptoms. A further cause had, therefore, to be sought, and it was then found that the trainer ordered at breakfast, luncheon, and before match practice, two or three glasses of fairly strong tea. Thus each man drank at least two quarts daily. Evidently some could endure it, the others could not. In any case, all symptoms disappeared within a week of the tea being withdrawn.

Caffeine and theine are undoubtedly useful stimulants on occasions, but abuse of their effect is easily produced, and in a variety of circumstances subacute intoxication may be encountered. It is well known that Alpine guides carry tea, urge its use, particularly at the rising meal, and for obvious reasons. In Russia tea is drunk more generally than in any other country, and particularly by all whose calling requires muscular effort. During the war there were many instances of troops carrying tea instead of water. Its use among aviators is also extensive. But the point for the physician to remember is that, stimulant though it is, there are sensitive human systems which can stand but relatively small quantities before the untoward or reactionary effects also begin to show.

### Chronic Tea Poisoning.

Far commoner than these acute conditions is simple chronic tea poisoning. That grave mental symptoms may possibly be caused by an abuse of tea has not been generally recognised, but in the reports of asylums in Ireland it is definitely classed as a cause of insanity.

The characteristic symptoms are described as insomnia, periods of despondency, alternating with states of anxiety and great restlessness. As the author admits, these may perhaps be ordinary maniacal depressive cases occurring among ordinary tea-drinking people, yet the influence of tea in their production has been repeatedly affirmed by competent men. The effect of tea in producing nervous symptoms certainly cannot be disregarded, and there are many depressant effects upon general health as well. A few years ago, also in the *Medical*

*Record*, a careful analysis of general health among domestic servants was published, and 10 per cent. of cases of ordinary ill-health among this class were claimed as traceable to tea. Often as many as twelve to fifteen cups were taken daily, and it is with certainty stated that, in the above percentage, withdrawal of all tea rapidly caused a disappearance of the complaints. Too often in our dispensaries such cases are dismissed with a mixture of rhubarb and soda, and the true cause is never suspected.

Prohibition?

There is, therefore, little doubt that, in excessive quantities, tea is to be classed as a poison. The facts are all against it in any quantities other than small, but, in justice to the other side of the question, one may say with Professor Starr that in tea-drinking, "There is a sense of increased power, and attended by a pleasurable sensation, a feeling of brightness, cheerfulness, and mental activity—not perhaps as rapidly developed or as intense as that produced by alcohol, but quite comparable. One hesitates to put this on record, lest a wave of tea prohibition be started. It is a result, however, which explains the growing, almost universal, use of tea, especially in the late afternoon, when fatigue, both physical and mental, of the day seems to make one desirous of an artificial stimulant. It would be foolish to assert that this is in any way harmful in moderation. . . . Study of the social use of tea leads us to the conclusion that much solace and satisfaction is to be obtained from the cup which cheers—but not inebriates."

